# Retrieval of intrarenal coiled and ruptured guidewire by retrograde intrarenal surgery: A case report and literature review

**DOI:** 10.1515/med-2021-0385

**Published:** 2021-11-12

**Authors:** Bo-Han Chen, Tsung-Hsin Chang, Marcelo Chen, Yu-Hsin Chen

**Affiliations:** Department of Urology, MacKay Memorial Hospital, Zhongshan District, Taipei, Taiwan; Department of Medicine, Mackay Medical College, New Taipei City, Taiwan; Institute of Pharmacology, School of Medicine, National Yang-Ming University, Taipei, Taiwan; Department of Urology, MacKay Memorial Hospital, No. 92, Sec. 2, Zhongshan N. Rd., Zhongshan District, Taipei, Taiwan

**Keywords:** intrarenal foreign body, retrograde intrarenal surgery, percutaneous nephrostomy drainage

## Abstract

**Background:**

Foreign bodies in the kidney have rarely been reported. However, they can be a clinical problem for urologists. We report on a patient with a residual segment of guidewire coating embedded in the renal parenchyma following computed tomography (CT)-guided percutaneous nephrostomy drainage (PCND), and our successful minimally invasive management with retrograde intrarenal surgery (RIRS).

**Case presentation:**

A 40-year-old female with urosepsis due to a right upper ureteral stone with hydronephrosis received emergent CT-guided PCND and subsequent ureteroscopic lithotripsy, double J stent insertion, and percutaneous catheter removal. Follow-up radiography showed a coiled object within the upper pole parenchyma of the right kidney, which might be the remnant of a guidewire used during the PCND procedure. Flexible ureteroscopy (fURS) was performed. Under fluoroscopy, the foreign body was localized, the renal parenchyma was incised with laser, and the foreign body was retrieved using a stone basket.

**Conclusion:**

Although guidewire breakage is uncommon, clinicians should keep it in mind during interventional procedures. Several methods can be used to eradicate foreign objects from the urinary tract, and the first choice should always be the least invasive one. RIRS with fURS is considered as a safe, efficient, and minimally invasive option for the extraction of foreign bodies from the kidney. To the best of our knowledge, this is the first comprehensive case report detailing the removal of a foreign object by RIRS in the English literature.

## 1 Introduction

Foreign bodies in the kidney have rarely been reported and it has seldom been elaborated in the urological literature [1]. However, foreign bodies in the urinary tract can be a clinical problem for urologists. Foreign bodies can be introduced to the kidney by penetration during a traumatic incident, by oral ingestion, and intestinal perforation, and sometimes even because of the accidental leaving of items in the body during medical procedures [2,3,4,5,6].

We hereby report a patient with a residual guidewire segment left behind in the kidney during computed tomography (CT)-guided percutaneous nephrostomy drainage (PCND), and our successful minimally invasive management with retrograde intrarenal surgery (RIRS). To the best of our knowledge, this is the first comprehensive case report detailing the removal of a foreign object by RIRS in the English literature.

## 2 Case presentation

A 40-year-old female who had a medical history of Sicca syndrome and regularly received follow-up at the rheumatology clinic, presented at our emergency department with right flank pain and a fever of up to 40° Celsius for several days. She had no history of trauma and there was no personal or family history of diabetes mellitus, urolithiasis, or malignancies. A physical examination revealed right costovertebral knocking tenderness. Urine examination revealed seven white blood cells per high-power field. Laboratory tests revealed leukopenia with a total white blood cell count of 2,300/μL (normal range: 4,000–10,000/μL) and an elevated C-reactive protein (CRP) level of 17.4 mg/dL (normal range: 0.0–0.79 mg/dL). Renal function test results were within normal ranges.

CT revealed a 1 cm right upper ureteral stone causing upper stream hydronephrosis and obstructive uropathy. The patient received emergent CT-guided PCND by radiologists and was admitted for infection control. After her infection was controlled, ureteroscopy with laser lithotripsy with a double J stent insertion was performed. The PCND tube was removed during the surgery and the patient was discharged the following day.

During her postoperative follow-up at our outpatient department, the patient still complained of right flank pain and gross hematuria off and on. She denied muscle strain, skeletal trauma, fever, dysuria, and changes in bowel habits. A physical examination revealed knocking tenderness at the right costovertebral angle. Laboratory tests were within the normal range including white blood cell count, CRP, and renal function test.

**Figure 1:**
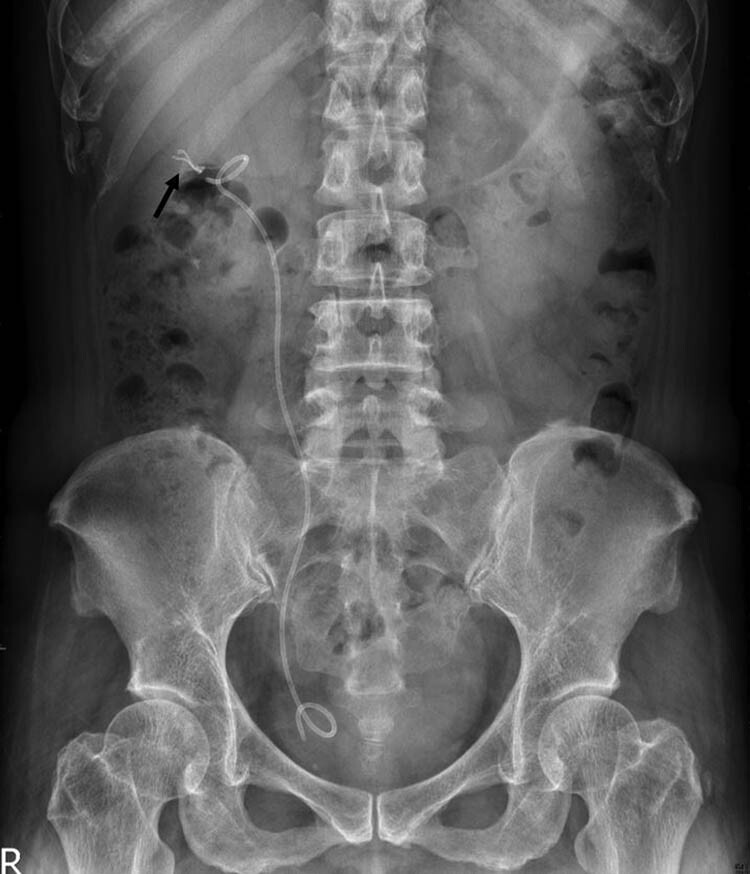
Post-PCND follow-up KUB show a new radiopaque lesion (arrow) in right kidney area.

**Figure 2:**
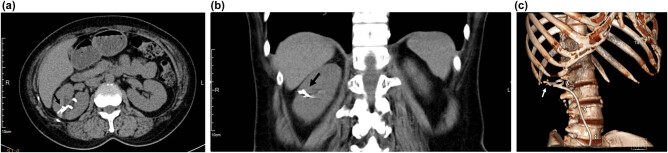
Computed tomography image of the intrarenal foreign body (arrow) and its relationship with right double J ureter stent (arrowheads). (a) Transverse cut, (b) coronal cut, and (c) reconstruction.

A plain radiograph of the kidneys, ureters, and bladder (KUB) showed a new radiopaque lesion in the right renal shadow compared with the pre-PCND plain film (Figure 1). This artifact was considered to be a retained ruptured guidewire or fragments of the PCND. Further CT imaging was performed to determine the precise anatomical location and the radiodensity of the object, which was revealed to be a curvilinear hyperdense coil within the upper pole parenchyma of the right kidney (Figure 2a–c). The radiologists judged that the coil could be the remnants of a guidewire used during the PCND procedure.

**Figure 3:**
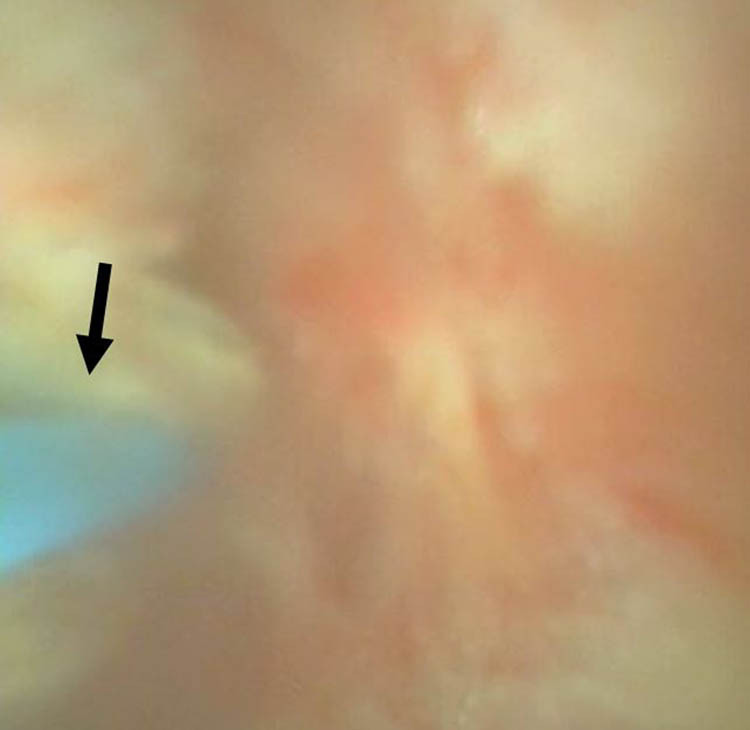
The video image of fURS and the laser probe (arrow) for cutting the underlying renal parenchyma for exposure of the embedded material.

**Figure 4:**
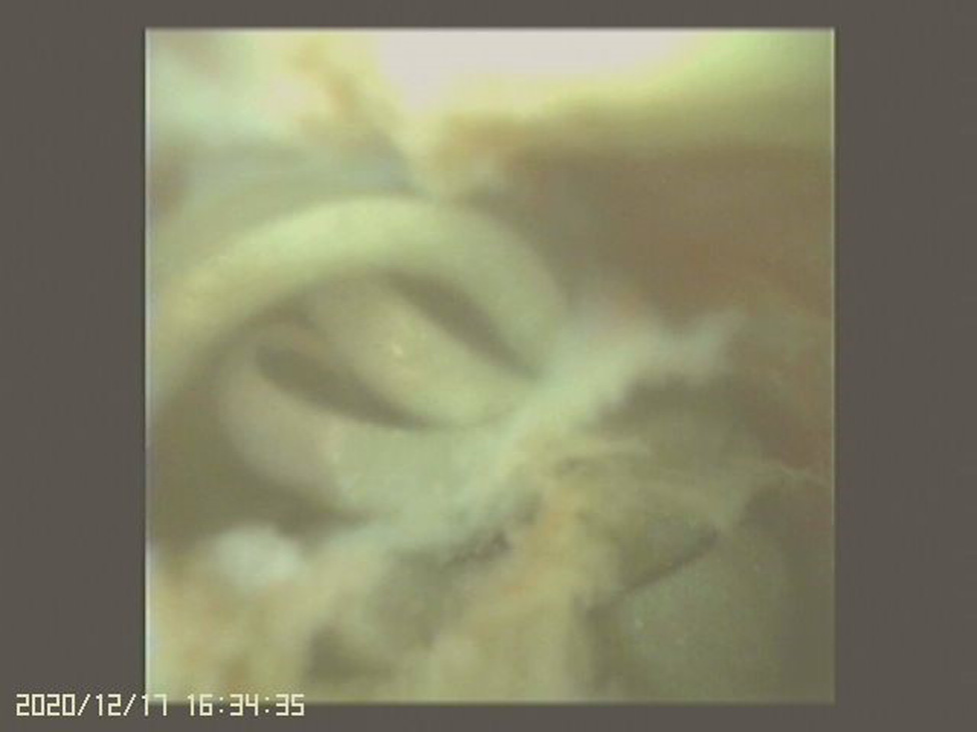
The video image of fURS and the coiled element.

**Figure 5:**
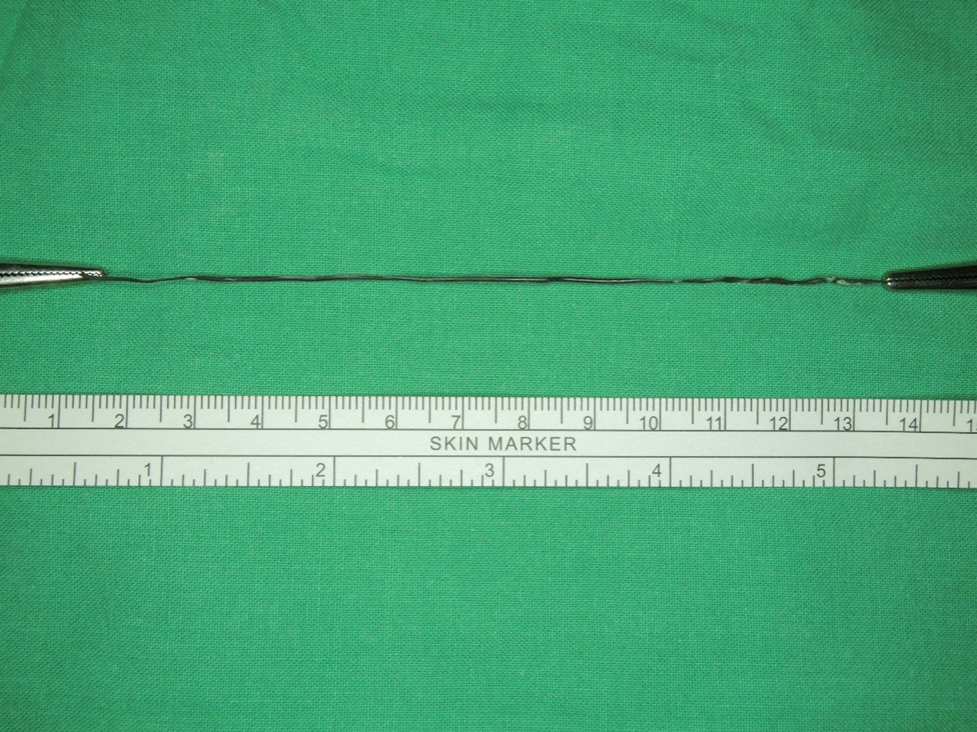
The retrieved specimen from RIRS (12 cm).

We therefore performed RIRS with flexible ureteroscopy (fURS) (SemiFlex Scope™) via a ureteral access sheath (Well Lead ClearPetra 11/13French–36 cm) under intraoperative C-arm fluoroscopy. There was no visible foreign body or attachment points within the pyelocalyceal system in the fURS field. We then located our fURS at a suspicious site under C-arm fluoroscopy guidance, which could be the location of the embedded coil within the renal parenchyma. To aim our target, we operated C-arm fluoroscopy not only in vertical axial but also in horizontal axial and at an angle of 45°. Unfortunately, the target could not be seen under direct ureteroscopic vision. We only identified a hint of a defect, which could have been the penetration site of the previous PCND procedure. A Holmium-YAG Laser (Lumenis® VersaPulse® PowerSuite™ 200 μm laser system, 1.5 J and 20 Hz) was used to cut through the pit defect and the underlying renal parenchyma to gradually expose the embedded foreign body (Figure 3). To prevent patient from bleeding during laser cutting, we kept in low power setting, which would cause less injury to the surrounding tissue. To avoid unnecessary injury to renal parenchyma, anesthesiologist helped to control the patient’s breath which could minimize the movement of her kidney during laser cutting. A coiled object was finally observed (Figure 4) and it was carefully retrieved using a Boston Scientific Zero Tip™ Nitinol stone basket. The specimen was a 12 cm long tube-like object (Figure 5), which was determined to be with the coating layer of a guidewire, which had broken during the previous CT-guided PCND. The procedure was completed by careful hemostasis and the placement of a 4.7 French–22 cm (Cook Medical) double J ureteral stent.

**Figure 6:**
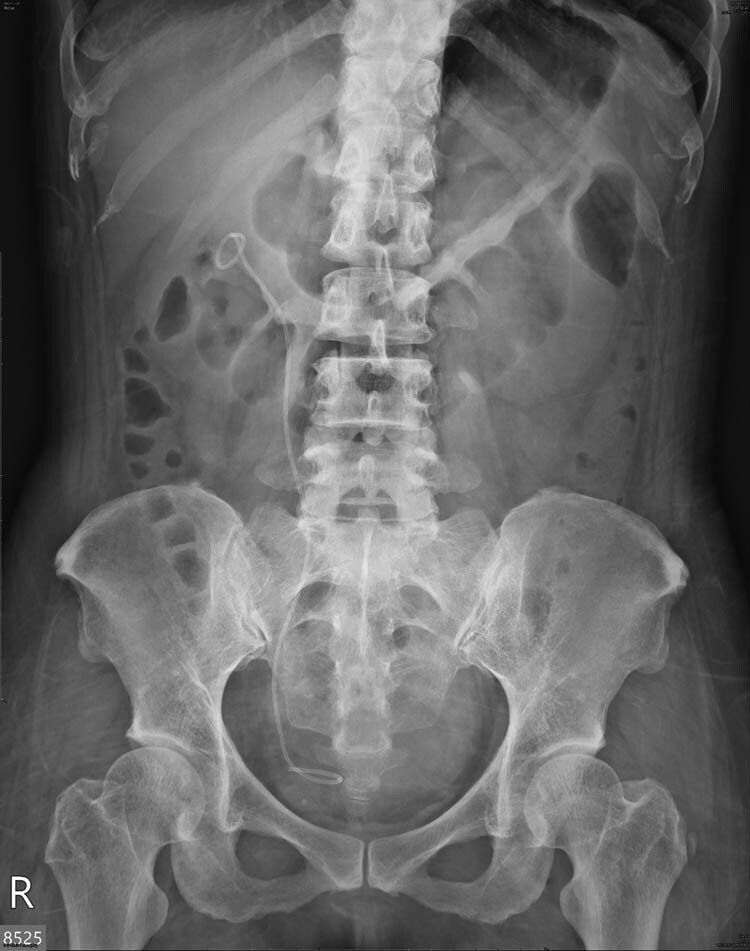
Postoperative KUB showed disappearance of the radiopaque lesion at right kidney area. Right double J ureteral stent were *in situ*.


Postoperative KUB revealed the disappearance of the radiopaque lesion in the right renal shadow (Figure 6). The patient’s symptoms also improved and neither flank pain nor gross hematuria was noted during follow-up. Three weeks after the procedure, the double J stent was removed without complications.

**Consent for publication:** Informed consent was obtained in both written and verbal format from the patient to publish this case report and any accompanying images.
**Consent for the treatment (Informed consent):** Informed consent has obtained from all individuals included in this study.

## 3 Discussion and conclusion

Foreign bodies in the kidney and renal pelvis are rarely reported; however, urologists should be reminded of their clinical symptoms and roentgen signs [7]. Symptoms related to foreign bodies vary between individuals; some may be troublesome due to irritative symptoms and some may be totally asymptomatic. Hematuria and flank pain are the most common symptoms, especially for upper urinary tract foreign bodies [6]. It is important to carefully remove all foreign body fragments whether they are symptomatic or not. It has been reported that foreign bodies can become a nidus for stone formation, and they can cause intractable bacterial infections [3,6,8,9]. A correct diagnosis and clear preoperative identification of the anatomical location of the foreign body can guide the clinical decision and can help urologists to minimize the potential harm to the patient.

Management can be challenging depending on the size, shape, mobility and location of the foreign bodies [6,9]. Furthermore, thorough history taking and optimal imaging are the most important factors during the preoperative evaluation. [9]. There have been some previously published case reports describing intrarenal foreign bodies. Manassero et al. demonstrated an open nephrectomy using an anterolateral extraperitoneal approach for the retrieval of an intrarenal device [4]. Sener et al. dissected the right ureter and excised a guidewire introducer foreign body, through a right iliac incision with a ureteroneocystostomy and Lich-Gregoire anastomosis [10]. Upadhyay et al. removed three encrusted metallic foreign bodies from the right kidney by nephroscopy through a 32 French nephrostomy tract [2]. Singh et al. performed a transperitoneal laparoscopic exploration and removed a 4.5 cm long malleable copper wire from the left renal parenchyma [1]. Tüdös et al. used a rigid ureterorenoscopy with a 8.5/11.5 French and grasping forceps to extract a wooden toothpick from the right renal pelvis [11].

In the present case, the guidewire fragments were tightly embedded in the renal tissue without extension into the pyelocalyceal system or to the outside of the renal parenchyma. Partial nephrectomy, percutaneous nephroscopy, semi-rigid ureteroscopy, RIRS with fURS, and observation were all potential choices for our patient. The intrarenal foreign body from our patient was similar to the device which was retrieved by Manassero et al. through an open nephrectomy [4]. However, the risks, including bleeding, infection, and injury to the renal tissue, associated with those more invasive procedures pose a threat to the patient and can potentially cause morbidity if not handled well. Since several methods can be used to eradicate foreign objects from the urinary tract, the first choice should always be the least invasive one. RIRS was the least invasive and therefore most appropriate choice for our case.

Iatrogenic-retained foreign body is an important issue for all urologists and radiologists, not only because of the physical problems it can cause the patient but also because it may result in legal action. Hennessey et al. summarized that the placement of a percutaneous nephrostomy and associated percutaneous procedures accounted for 35% of all upper tract retained foreign objects [12]. More attention must be paid to avoid these unnecessary consequences. Careful examination of the instruments before and after procedures is essential and could decrease the incidence of such unwanted complications [13].

In conclusion, retained foreign bodies in the urinary tract are uncommon; however, clinicians must still keep them in mind during percutaneous procedures. Although there are several methods for eradicating foreign objects from the urinary tract, the least invasive method that does the least harm to the patient should be the first choice. RIRS with fURS is considered a safe, efficient, and minimally invasive option for the extraction of foreign bodies from the kidney.

## Abbreviations

CRP: C-reactive protein
CT: computed tomography
fURS: flexible ureteroscopy.
KUB: kidneys, ureters, and bladder
PCND: percutaneous nephrostomy drainage
RIRS: retrograde intrarenal surgery
